# Magnitude estimation and ground motion prediction to harness fiber optic distributed acoustic sensing for earthquake early warning

**DOI:** 10.1038/s41598-023-27444-3

**Published:** 2023-01-09

**Authors:** Itzhak Lior, Diane Rivet, Jean-Paul Ampuero, Anthony Sladen, Sergio Barrientos, Rodrigo Sánchez-Olavarría, German Alberto Villarroel Opazo, Jose Antonio Bustamante Prado

**Affiliations:** 1 Institute of Earth Sciences, The Hebrew University, Jerusalem, Israel; 2 Université Côte d’Azur, Observatoire de La Côte d’Azur, CNRS, IRD, Géoazur, Valbonne, France; 3 Centro Sismológico Nacional, Universidad de Chile, Santiago, Chile; 4 Gtd Grupo S.A., Santiago, Chile

**Keywords:** Seismology, Geophysics, Natural hazards

## Abstract

Earthquake early warning (EEW) systems provide seconds to tens of seconds of warning time before potentially-damaging ground motions are felt. For optimal warning times, seismic sensors should be installed as close as possible to expected earthquake sources. However, while the most hazardous earthquakes on Earth occur underwater, most seismological stations are located on-land; precious seconds may go by before these earthquakes are detected. In this work, we harness available optical fiber infrastructure for EEW using the novel approach of distributed acoustic sensing (DAS). DAS strain measurements of earthquakes from different regions are converted to ground motions using a real-time slant-stack approach, magnitudes are estimated using a theoretical earthquake source model, and ground shaking intensities are predicted via ground motion prediction equations. The results demonstrate the potential of DAS-based EEW and the significant time-gains that can be achieved compared to the use of standard sensors, in particular for offshore earthquakes.

## Introduction

While earthquake prediction remains out of reach, continuous seismic monitoring has enabled earthquake early warning (EEW) systems that provide alerts to population centers and critical infrastructure seconds to tens of seconds before intense ground shaking is felt^1–4^. Following rupture initiation, warning may be issued by analyzing recorded ground motions in real-time to assess the earthquake’s damage potential. The performance of EEW systems largely depends on the spatial distribution of available seismic sensors^5^; for fast and robust warning issuance, seismic instruments should be installed in proximity to active faults, where earthquakes are expected to occur. While most of the largest and most hazardous earthquakes on Earth occur offshore in subduction zones, the vast majority of seismic stations are located on-land. Thus, valuable time may be lost waiting for seismic waves to reach on-land stations^6^. Current solutions, such as densifying on-land seismic networks and installing cabled ocean bottom sensor networks, are implemented in Japan^7^ and Canada^8^. However, high costs preclude their worldwide implementation. An alternative is to convert existing fiber optic cables into dense seismic networks via the novel technology of distributed acoustic sensing (DAS)^9,10^. The ever-growing worldwide deployment of optical fiber telecommunication infrastructure, in particular submarine cables, opens opportunities for widespread low-cost implementation of DAS for EEW, circumventing costly ocean-bottom deployments and operations. The potential of seafloor DAS for EEW has not been quantitatively demonstrated yet, a gap addressed in this work.

Over the past several years, the unique advantages of DAS have proven valuable for various seismological purposes including earthquake analysis^11–13^ and subsurface imaging^14–17^. DAS enables the measurement of transient ground deformations (strains or strain-rates) along optical fibers (e.g., internet cables), such as those currently traversing most of our planet, both on-land and underwater. Unlike point-sensors (e.g., seismometers, accelerometers, GNSS), DAS yields spatially dense longitudinal deformation measurements (every several meters, typically 10) along tens-of-kilometers long optical fibers with a maximum range between 80 and 150 km, depending on the specific DAS interrogator. This technology allows for continuous monitoring of large regions and provides a more complete picture of the seismic wave-field. Measurements are obtained using an interrogator unit which is placed at one end of the cable and sends laser pulses along the fiber. Due to small heterogeneities within the fiber, a fraction of the transmitted light is backscattered via Rayleigh scattering. When seismic waves perturb the cable, heterogeneities along the fiber change positions, and so does the Rayleigh backscattering pattern. The backscattering phase differences between time samples are then translated into strain or strain-rate measurements at spacings of several meters along tens-of-kilometers long fibers^18^. This technique allows for the transformation of any optical fiber into a dense array of seismo-acoustic sensors, producing measurements with unprecedented spatial and temporal resolutions.

DAS has key features that are ideally suited for the challenges of EEW. It facilitates spatially and temporally continuous seismic measurements at hard-to-reach places, such as underwater^19^ and in boreholes^20^, closer to earthquake hypocenters. The dense spatial sampling facilitates more reliable separation between earthquakes and noise^21^ compared to point-sensors. Furthermore, the DAS interrogator is sensing the whole fiber from one of its ends, nullifying power and telemetry considerations to distant fiber segments. Thus, the use of optical fibers as dense seismic networks could be decisive in the performance of EEW systems, significantly improving earthquake warning times and allowing for better preparedness for intense shaking.

While the advantages of DAS for EEW are appealing, this novel technology suffers several drawbacks that need to be addressed. For DAS strain measurements to represent ground deformations, fibers should be adequately coupled to the ground. However, since many fibers were not deployed with seismological applications in mind, coupling can vary, and be insufficient along specific sections, for reliable measurements. State-of-the-art DAS interrogator units can sense fibers of up to ~ 150 km (as demonstrated by the earthquake recording performed by an Alcatel OptoDAS interrogator unit in Supplementary Fig. 1) or up to the first repeater, such that more than one system and fiber may be needed to cover vast regions. DAS records strains or strain-rates: these measurements are very sensitive to the local velocity structure beneath the fiber^13^ and to lateral subsurface heterogeneities^12,22^. This property is troublesome to both earthquake magnitude estimation, which typically relies on ground motion (i.e., displacements, velocities or accelerations) measurements^23^, and for earthquake location efforts, where sensitivity to the local subsurface structure may complicate earthquake sources’ locations^12^. In addition, DAS measures strains along the fiber’s axis, such that P-waves recorded by horizontal fibers typically induce low amplitudes that may even be below instrumental noise levels, potentially hindering earthquake location capabilities. Furthermore, while EEW is intended for large earthquakes at short distances, where damages are expected to occur, such DAS observations are currently unavailable. Thus, the effects of DAS amplitude saturation and cable-ground coupling behavior during intense deformations are insufficiently reported and studied. In the following sections we tackle several of the mentioned disadvantages, yet additional work is needed to address all issues to reliably use DAS for EEW.

In this work, we propose the first quantitative real-time schemes that will be part of an operational DAS-based EEW system. Early warning is typically achieved by (1) detecting an earthquake, (2) determining its location, (3) resolving the earthquake source parameters (magnitude and stress drop^24^), and (4) predicting ground shaking intensities, typically peak ground velocities (PGV) and peak ground accelerations (PGA)^25^. To the best of our knowledge, these four real-time objectives are yet to be addressed using DAS. Real-time earthquake detection and location may be achieved using either well-established point-sensor-based approaches^26–29^ applied to single or multiple DAS channels, or array processing techniques such as beamforming^12,30,31^. While detection can be achieved with relative ease even with point-sensor -based algorithms^27,29^, earthquake location poses several challenges that are unique to DAS data^12,22^. The recorded strain wavefield may not be coherent enough for reliable earthquake location or may be dominated by scattered waves. In addition, the geographical locations of measurements along the fiber need to be calibrated to reduce earthquake location errors. These issues will be considered when devising real-time earthquake location schemes, a subject of subsequent manuscripts. Here, we address the last two objectives: real-time magnitude estimation and shaking intensity prediction.

Most operational EEW systems rely on empirical relations for both magnitude estimation and ground motion prediction^32,33^. The robustness of these relations largely relies on the quality, quantity, and magnitude range of available earthquake observations^34^. Since DAS is a relatively new seismic measurement technology^9^, current earthquake DAS datasets are insufficient in all aspects to devise robust empirical methods, and a physics-based approach that does not rely on data availability should be developed^24,35,36^. Recently, a holistic physics-based approach for real-time earthquake source parameter (magnitude and stress drop) estimation and ground motion prediction has been proposed^24^. A similar method, adapted to DAS data, is developed here by deriving a theoretical expression for real-time magnitude estimation using the root-mean-squares (rms) of ground accelerations.

Since DAS measures strains (or strain-rates) and earthquake magnitude is directly related to ground motions (displacements, velocities and accelerations)^23^, DAS measurements first need to be converted to ground motions^37^. This objective is typically achieved by using the apparent slowness (reciprocal of velocity), ${p}_{x}$, measured along the fiber^38^:

1 $$\frac{{d^{n} }}{{dt^{n} }}D\left( t \right) = \frac{{d^{{n - 1}} }}{{dt^{{n - 1}} }} \epsilon \left( t \right)/p_{{x^{\prime } }}$$

where *ϵ*(*t*) and *D*(*t*) are the time-series of strains and ground displacements, respectively, and *n* equals 0, 1 or 2 corresponds to conversions to ground displacements, velocities or accelerations, respectively. This relation assumes perfect coupling between the fiber and the Earth, an assumption that was found to hold well for different fibers and installations^11,13,37,37–39^. Slowness has been observed to change rapidly both in time and space (along the fiber): temporal variations are due to velocity differences among recorded seismic phases (i.e., P-, S-, surface-waves)^37^ and spatial variations are a result of lateral subsurface-velocity heterogeneities, that may be significant and abrupt^12,16,22,37^. Accurate conversion of strain-rates to ground accelerations requires that slowness be resolved as a function of both time and space. Recently, a slant-stack based strains to ground motions conversion method has been proposed^37^, and is modified and adapted here for real-time processing.

The approaches presented in this manuscript build on the above-mentioned advancements in physics-based EEW^24^ and DAS earthquake data processing^37^. The potential of the modified strains to ground motions conversion and the new magnitude expression for EEW are examined in conjunction with a theoretical ground motion prediction equation (GMPE)^40^. In the following sections, we present and validate a computationally efficient real-time protocol that relies on straightforward analytical formulations for the analysis of DAS recorded earthquakes. Strain-rates are converted to ground accelerations using a real-time adapted slant-stack approach. Then, earthquake magnitudes are estimated via an analytical expression derived using the Omega-squared source spectra model^41,42^. Subject to the theoretical model, this magnitude expression is applicable to all body wave (P- and S-waves) far-field ground motion recordings. This scheme is applied to several well-coupled fiber segments along different ocean-bottom fibers. Finally, the magnitude is used to predict PGV and PGA away from the hypocenter using a GMPE^24,40^, derived using the same Omega-squared source model. The fact that both magnitude estimation and ground motion prediction are derived from the same theoretical model contributes to the stability and consistency of the estimates, as shown in the next sections. Magnitude and peak ground shaking predictions are continuously updated and modified as new seismic signals are recorded. We demonstrate the robustness of these real-time approaches for a wide magnitude range and show that using well-coupled offshore fibers for EEW can significantly improve warning times compared to those expected from standard point-sensor-based EEW systems.

To demonstrate the merits of the proposed DAS-based EEW schemes, we compiled a DAS earthquake dataset from different tectonic environments. Data were recorded by four different ocean-bottom fibers: two offshore Greece^13,16,37,43^, one offshore France^13,19,37,44^ and one offshore Chile (Supplementary Fig. 2). The measurements in Greece, France and Chile were conducted using three different interrogator units: a Febus A1 DAS interrogator, an Aragon Photonics hDAS interrogator and an ASN OptoDAS interrogator, respectively (See “Earthquake dataset” in Methods). We analyzed a total of 53 DAS recorded earthquakes that range from magnitude 2 to 5.7 (Supplementary Fig. 2) at hypocentral distances of 17 to 365 km (Supplementary Fig. 3). Earthquake metadata are provided in Supplementary Table 1.

## Results

### Using DAS data for magnitude estimation

**Figure 1:**
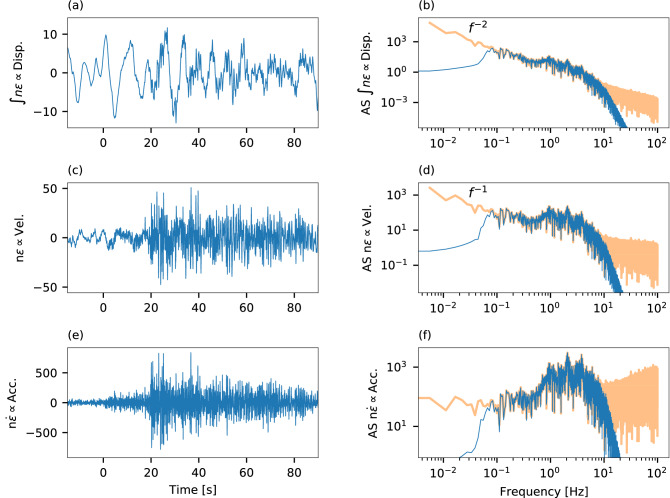
DAS instrumental noise. (**a**,**b**) Strains-integral, (**c**,**d**) strains, and (**e**,**f**) strain-rates of a magnitude 3.6 earthquake recorded at a hypocentral distance of 135 km at 21 km along a fiber offshore Greece. Signals filtered between 0.06 and 10 Hz are shown on the left as a function of time from P-wave arrival (**a**,**c**,**e**) and the corresponding spectra are shown on the right (blue curves **b**,**d**,**f**). The prefiltered spectra (orange curves **b**,**d**,**f**) demonstrate that low frequency noise is **b** ∝ *f *^−2^, **d** ∝ *f *^−1^, and **f** independent of frequency.

Ideally, moment magnitude should be estimated using seismic recordings of ground displacements, *D*, rather than ground velocities, *V*, or accelerations, *A*, and the signals should include as much of their low-frequency portion as possible to avoid magnitude saturation^23,35^. Ground displacements can be obtained from well-coupled DAS measurements by integrating strain measurements in time (or double integration of strain-rates) and dividing them by the apparent slowness (n = 0 in Eq. 1)^11,37–39^. This conversion approach has been demonstrated by previous studies that considered DAS instrument response^11^ and coupling^45^. However, the use of DAS converted ground displacements is challenging given the inherently high instrumental noise levels, especially at large distances along long fibers^13,46,47^. The behavior of DAS instrumental noise is demonstrated in Fig. (1) for an earthquake of magnitude 3.6 recorded at a distance of 135 km from an optical fiber offshore southeastern Greece^13,16,37,43^ (See map in Supplementary Fig. 2). At low frequencies, the instrumental noise of the time-integral of strains (∝ *D*, Fig. 1a,b), strains (∝ *V*, Fig. 1c,d) and strain-rates (∝ *A*, Fig. 1e,f) is proportional to *f *^−2^, *f *^−1^ and independent of frequency, *f*, respectively. As a result, strains-integral (∝ *D*, Fig. 1a) and strains (∝ *V*, Fig. 1c) time-series are contaminated by low-frequency noise, and their use may lead to magnitude over-estimation and false alarms. Thus, we only use strain-rates (∝ *A*) for real-time magnitude estimation even though they present a weaker correlation with earthquake magnitude compared to strains-integral (∝ *D*) and strains (∝ *V*) (See “The relation between earthquake source parameters and ground motions” in Methods). Since strain-rates’ instrumental noise increases as *f* at high frequencies (Fig. 1f), a lowpass filter is needed. This filter will not bias magnitude estimations because larger earthquakes produce lower frequency radiation.

### Strain-rates to ground accelerations conversion

**Figure 2:**
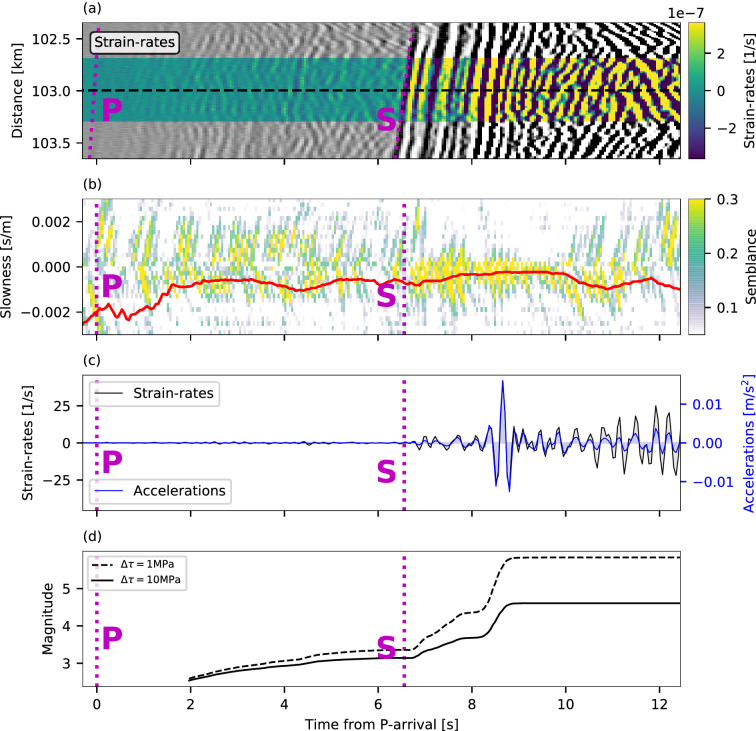
Strain-rates to ground accelerations conversion and magnitude estimation. **a** Strain-rates of a magnitude 3.8 earthquake recorded at a hypocentral distance of 60 km between 102.3 km and 103.6 km along a fiber offshore Chile. The fiber segment used for magnitude estimation is color-coded (102.7 km to 103.3 km). **b** Semblance as functions of apparent slowness and time from P-wave arrival for a reference DAS channel at 103 km from the interrogator (black dashed line in **a**). Smoothed slowness (See Methods) is indicated by a red curve. **c** Strain-rates (black) and converted ground accelerations (blues) for the reference DAS channel. **d** Real-time magnitude evolution using stress drops of 1 MPa (dashed curve) and 10 MPa (solid curve). Manually picked P- and S-wave arrival times are indicated by magenta lines.

The performance of the conversion algorithm (See “Real-time strain-rates to ground accelerations conversion” in Methods) is demonstrated for a magnitude 3.8 earthquake recorded offshore Chile at a hypocentral distance of 60 km (See map in Supplementary Fig. 2) for a single DAS channel at a distance of 103 km along the fiber (Fig. 2). Note that direct P-waves are not visible, although P-wave induced scattered-waves are clearly seen (1–6 s in Fig. 2a,b). The same analysis for the largest earthquake in the dataset, a magnitude 5.7, is shown in Supplementary Fig. 4; for this earthquake, strain-rate amplitudes exhibit some saturation (See Discussion). The real-time slant-stack approach resolves the apparent velocities of the different seismic phases: ~ 4.2 km/s for direct S-waves (6–9 s in Fig. 2a,b) and ~ 1.8 km/s for surface-waves (e.g., 1–6 s and 10–13 s in Fig. 2a,b). Owing to these velocity variations, ground accelerations are somewhat different from strain-rates: accelerations (blue curve in Fig. 2c) exhibit a noticeable amplitude difference between fast S-waves and slow surface-waves, while strain-rates (black curve in Fig. 2c) display similar amplitudes for both phases. A comparison between the performance of the real-time slant-stack conversion and the previously presented approach^37^ indicates that the real-time adaptations do not decrease the conversion quality (Supplementary Fig. 5).

### The effect of stress drop variability

**Figure 3:**
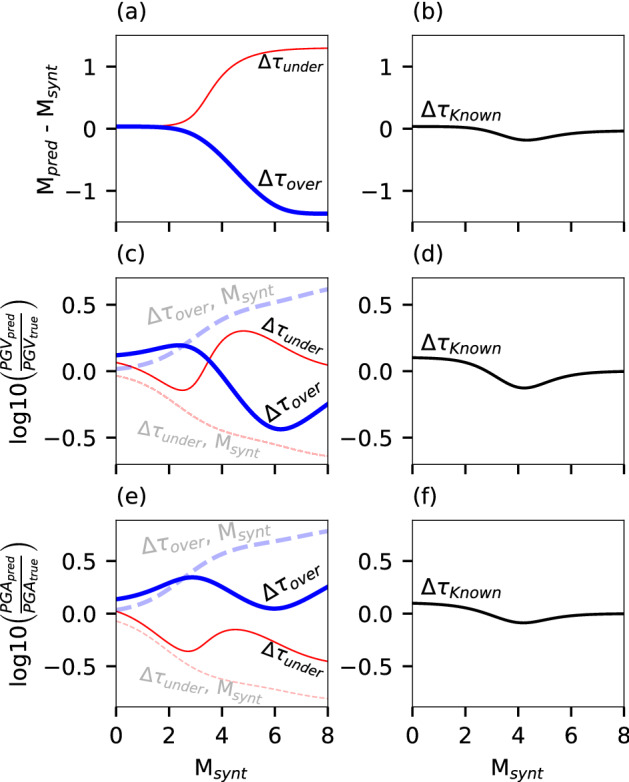
The effect of stress drop variability on magnitude estimation and ground motion prediction. Estimated minus synthetic magnitude as a function of synthetic magnitude for (**a**) under-estimated (1 MPa), over-estimated (100 MPa) and (**b**) known (10 MPa) stress drops. The logarithms of predicted peak ground motions minus the logarithms of synthetic peak ground motions as functions of synthetic magnitude are shown for PGV for (**c**) 1 MPa and 100 MPa and (**d**) 10 MPa, and for PGA for (**e**) 1 MPa and 100 MPa and (**f**) 10 MPa. The effect of using synthetic magnitude on (**c**) PGV and (**e**) PGA discrepancies is indicated by semi-transparent dashed curves. In all panels, curves corresponding to 1, 10 and 100 MPa are indicated by red, black and blue curves, respectively.

Stress drop, Δτ, is a fundamental earthquake source parameter that strongly affects ground motion intensities^40,48–50^ (See “The relation between earthquake source parameters and ground motions” in Methods). For optimal ground motion prediction, both magnitude and stress drop should be determined, as demonstrated by recent studies^3,36,40^. Since in this framework we only use one ground motion metric, i.e., ground accelerations rms, *A*_*rms*_, we may only estimate the magnitude (see “Magnitude estimation from bandlimited ground accelerations” in Methods) while the stress drop needs to be set a priori. Because *A*_*rms*_ are highly affected by the stress drop, and because its a priori value may deviate from its earthquake-specific real value^40,51^, it is useful to examine the effect of stress drop variability on magnitude estimation and intense shaking prediction. To this end, we synthesized *A*_*rms*_ using an ideal lowpass Butterworth filter, and PGV_*synt*_ and PGA_*synt*_ for different magnitudes using Δτ = 10 MPa at a hypocentral distance of 50 km (See “Synthetic ground motions” in Methods). We then used the synthetic *A*_*rms*_ to estimate the magnitudes, using different a priori stress drops of 1, 10 and 100 MPa (Eq. 7). The estimated magnitude and a priori stress drop were then used to predict PGV_*pred*_ and PGA_*pred*_ (Eq. 10), assuming that the distance is known (Fig. 3). When using Δτ = 10 MPa in Eqs. (7) and (10), magnitude, PGV, and PGA discrepancies are small (panels b, d and f of Fig. 3, respectively) and mostly attributed to the approximations made in deriving Eq. (7) (See Supplementary Note 1). When the stress drop in Eq. (7) is under-estimated (Δτ = 1 MPa) and over-estimated (Δτ = 100 MPa), magnitudes are over-estimated and under-estimated, respectively, by as much as 1.33 magnitude units for large earthquakes (Fig. 3a). When these biased magnitudes and stress drops are used to predict PGV, and PGA, they result in reasonable predictions: the standard deviations of the residuals are limited to ~ 0.43 log_10_(PGV) and log_10_(PGA) units (solid curves in Fig. 3c,e, respectively). This behavior is explained by inspecting Eq. (10): to first order^40^, $PGV\propto {M}_{0}^{1/2}\Delta {\tau }^{1/2}$ and $PGA\propto {M}_{0}^{1/3}\Delta {\tau }^{2/3}$, so using under-estimated stress drops along with over-estimated seismic moments (and vice-versa), as is the case here, will result in relatively small PGV and PGA discrepancies; Magnitude and stress drop biases reduce each other’s effect on ground motion predictions. In contrast, if synthetic magnitudes are used in conjunction with the over- and under-estimated stress drops, PGV and PGA discrepancies would be significantly higher (dashed curves in Fig. 3c,e). Further explanations on the shape of the residual plots are provided in Supplementary Note 2.

When implementing the proposed methods to different fibers in different tectonic settings, a priori stress drop may be estimated using available earthquake observations^40,52,53^ or taken from previous studies, if available. However, the results in Fig. 3 show that while the discrepancies between the synthetic earthquake stress drop and that used in Eq. (7) may have a significant impact on magnitude estimation, the effect on ground motion prediction is minimized, and the approach may be reliably used even with a biased stress drop. The effect of stress drop variability will be further examined using recorded earthquakes in the following section.

### Real-time magnitude estimation and peak ground shaking prediction

The performance of the real-time strain-rates to ground accelerations conversion, magnitude estimation, and ground motion prediction are demonstrated using a composite earthquake catalog of 53 DAS and point-sensor (seismometer and accelerometer) recorded earthquakes from Greece, France, and Chile (See “Earthquake dataset” in Methods, earthquake catalog in Supplementary Table 1, and maps showing the locations of earthquakes, fibers, and point-sensors in Supplementary Fig. 2). These earthquakes range from magnitude 2 to 5.7 (Supplementary Fig. 3) and were recorded by four different offshore fibers using three different DAS interrogators. DAS records are converted to ground accelerations and used to estimate the magnitude, while point-sensor records are used to compare observed and predicted PGV and PGA. Earthquake locations (and hypocentral distances) and P- and S-wave arrival times are assumed to be known: the former are extracted from available earthquake catalogs and the latter are manually picked. In practice, earthquake location and phase picking will be achieved in real-time via additional modules, whose development is beyond the scope of this manuscript. Thus, the uncertainties and discrepancies reported in this section are expected to be larger when earthquake detection and location are also implemented in real-time.

As previously stated, the goal of an EEW system is to produce robust ground motion predictions, while magnitude estimations are merely a by-product. In addition, while we estimate moment magnitudes, most catalogs report local magnitudes, whose values may significantly differ^54^. Thus, in subsequent analysis, we focus on the discrepancies between predicted and observed PGV and PGA as a measure for the algorithms’ performance, and provide less attention to the agreement between real-time and catalog reported magnitudes.

**Figure 4:**
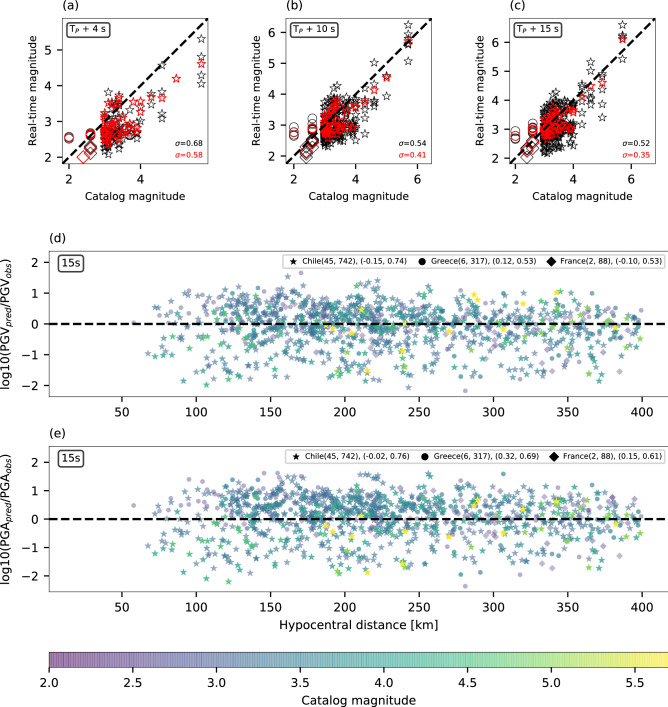
Real-time magnitude estimation and ground motion prediction using 10 MPa. Real-time magnitude as a function of catalog magnitude at **a** 4, **b** 10 and **c** 15 s from the P-wave arrival at the first fiber segment. Fiber-segment-specific estimates and event averages are indicated by black and red symbols, respectively. The dashed black line is a 1:1 line and the standard deviations of the magnitude residuals are indicated in the bottom right corners for segment specific (black) and event averaged (red) estimates. Discrepancies between the logarithms of predicted and observed peak ground motions are plotted for **d** PGV and **e** PGA as functions of hypocentral distance. Color-code corresponds to catalog magnitudes. Earthquakes from Chile, Greece and France are indicated by stars, circles and triangles, respectively. Panel legends indicate the following: cable name (number of earthquakes, number of PGV and PGA observations), (average residuals, standard deviation to the residuals). Average within event variabilities, i.e., the optimal standard deviation to the residuals, for PGV are 0.68, 0.5 and 0.52 for Chile, Greece and France, respectively, and for PGA are 0.71, 0.61 and 0.59 for Chile, Greece and France, respectively.

Magnitude is estimated using several well-coupled fiber segments for each cable. Coupling quality is evaluated by inspecting earthquakes’ seismic wavefield along the fiber and identifying sections that display continuous seismic wavefronts and small amplitude variabilities of less than 4 dB^37^. DAS data are converted to ground accelerations and an initial magnitude estimate is obtained two seconds following P-wave detection at the first fiber segment, and is continuously updated with increasing data intervals and as the earthquake is recorded at additional locations along the fiber. The analysis uses all available phases including direct P- and S-arrival, scattered waves and surface waves. For P-waves, which are seldom undetected by horizontal DAS arrays, scattered and later arriving P-phases are used for the analysis. In this work, phases were identified and picked manually, while in real-time it will be achieved via automatic algorithms^21,31^. Real-time magnitude estimation is demonstrated for a catalog magnitude 3.8 earthquake using a single fiber segment in Fig. 2d. Magnitude increases with time, starting at the scattered P-waves (2–7 s), followed by a significant increase with the S-wave arrivals (7–9.5 s). As theoretically predicted (Fig. 3), magnitude estimates vary for different a priori stress drops, with magnitudes of 5.8 and 4.6 for 1 and 10 MPa, respectively, at 9.5 s from P-wave detection. Similar behavior is observed for the catalog Magnitude 5.7 earthquake shown in Supplementary Fig. 4d. Magnitude estimates improve with time as seen in Fig. 4a–c where real-time and catalog magnitudes are compared at 4, 10 and 15 s from the first P-wave detection, for the entire dataset.

A comparison between predicted (Eq. 10) and observed (See “Earthquake dataset” in Methods) PGV and PGA at 15 s indicates that the residuals are independent of hypocentral distance (Fig. 4d,e and Supplementary Fig. 6d, e) and catalog magnitude (Supplementary Fig. 7d, e), and that their standard deviations are relatively small, only slightly higher than the optimal values, i.e., within-event variabilities reported in the caption of Fig. 4. The latter result suggests that peak ground motion residuals are mainly caused by different site and path conditions that may be accounted for in future implementations, subject to additional research. While magnitude estimates are highly sensitive to the a priori stress drop, PGV and PGA residuals exhibit low sensitivity (Fig. 4 and Supplementary Fig. 6 for 10 and 1 MPa, respectively). This behavior is further demonstrated by examining the average magnitude, and PGV and PGA residuals for the largest available earthquake (Supplementary Fig. 8): average residuals show little sensitivity to stress drop and similar trends to those theoretically predicted (Fig. 3), i.e., PGV residuals are higher for stress drop under-estimation, and PGA residuals are lower for stress drop under-estimation.

## Discussion

**Figure 5:**
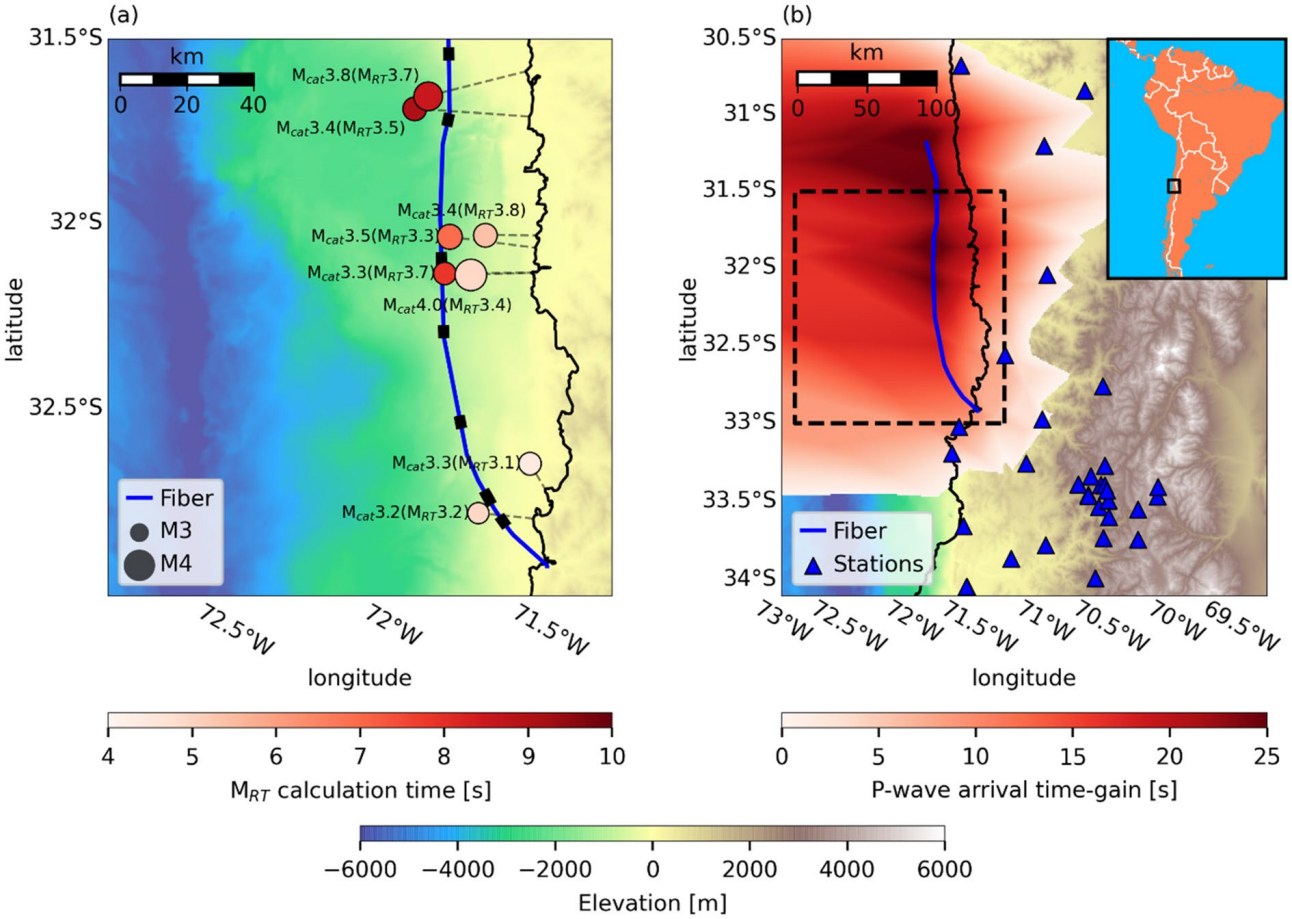
Time-gain using DAS offshore Chile. The region is indicated by a black rectangle in the inset map in **b**. **a** Catalog (M_cat_) and real-time (M_RT_) magnitude estimates when S-waves are expected to reach the coastline. Earthquakes are indicated by circles with size corresponding to catalog magnitudes, and color corresponding to M_RT_ estimation times. The shortest path to the coastline is indicated by grey dashed lines for each earthquake. The fiber is indicated by a blue curve and fiber segments used for magnitude estimation are indicated by black rectangles. **b** P-wave time-gain (red color scale) for different possible earthquake locations. Only positive time-gains are shown; negative time gains indicate earthquake locations that are closer to the fourth closest point-sensors than to the fiber. Point-sensors are indicated by blue triangles. The region shown in **a** is indicated by a black dashed rectangle in **b**. Maps were generated using Python’s Basemap package and bathymetric data downloaded from ncei.noaa.gov/maps/bathymetry/.

The results presented in this manuscript demonstrate that DAS can be reliably used for real-time magnitude estimation and ground motion prediction, two critical components of an operational EEW system. The use of DAS for EEW presents several significant advantages compared to the use of standard point-sensors, especially in the time-gain for offshore earthquakes. This latter advantage is illustrated in Fig. 5 using the fiber deployed offshore Chile, where ocean-bottom earthquakes pose a significant seismic hazard. For the offshore earthquakes shown in Fig. 5a, by the time S-waves are expected to reach the Chilean coastline, real-time magnitude estimates are typically within half a magnitude unit of catalog values, allowing for robust alert issuance before intense ground shaking is felt onland, and well before earthquakes are recorded by the available seismic network (Fig. 5b). The time-gain achieved by using the offshore Chile fiber compared to the current point-sensor network is defined here as the difference between the P-wave arrival at the closest fiber segment and at the fourth seismic station, as commonly required by EEW systems^55^. This time-gain may be as large as 25 s for earthquakes that occur near the fiber and may even result in early detection and alert issuance for onland earthquakes where point-sensor coverage is sparse (Fig. 5b). These precious seconds can have a decisive impact on mitigating the risk posed by potentially catastrophic offshore earthquakes.

Together with the time-gain for offshore earthquakes, DAS-based EEW can potentially outperform point-sensor-based EEW for several reasons. When implemented on well-coupled fiber segments, magnitude estimates are more reliable since data from many closely spaced DAS channels are averaged, reducing the impact of outliers and smoothing local effects. DAS facilitates robust differentiation between earthquakes and noise since earthquakes’ seismic wavefield is near-instantaneously recorded on hundreds-of-meters long fiber segments. As a result, false detections will be reduced and one fiber segment may be sufficient to issue early warning, subject to earthquake location capabilities.

While direct S-waves are detected by horizontally installed fibers, direct P-waves are usually not (Fig. 2a), a result of their fast velocities and the angle between the waves’ polarization and the fiber’s axis^13,56^. In contrast, P-wave induced scattered-waves are well recorded (2–6 s in Fig. 2a,b). Direct P-waves, if available, and S-waves, as well as scattered P- and S-waves are all used for magnitude estimation. While scattering results from heterogeneities of Earth’s media and varies from one region to another, the use of the waves’ apparent velocities to convert strain-rates to ground accelerations reduces the effect of this local phenomena on magnitude estimations. The dominance of these scattered waves will pose difficulties for earthquake location, since using scattered P-waves instead of direct P-waves will likely point to the scatterers’ locations rather than the earthquake’s source. Because P-wave based magnitudes are typically under-estimated (Fig. 2d), they are not expected to cause false alarms, yet they may be sufficient to surpass predefined alert thresholds. Since for EEW, sensors should be placed at proximity to expected epicenters, the closer the fiber is to earthquake locations, the sooner the high signal-to-noise S-waves are detected and used. For large earthquakes, the lower sensitivity of DAS to P-waves is an advantage because it will limit the saturation of direct and scattered P-waves.

Since DAS is an emerging technology, available datasets do not contain sufficient earthquakes whose damage potential is of interest for EEW. As a result, several technical aspects of the proposed schemes such as amplitude saturation and fiber-ground coupling during strong shaking cannot be fully evaluated. However, unlike commonly used empirical EEW approaches^32–34^, the proposed scheme is theoretical and relies on a well-established source model^41^ that was found to adequately describe the far-field radiation of a large range of earthquakes. Thus, showing that the proposed methods work for the current earthquake dataset is sufficient to demonstrate their validity. In addition, considering small magnitude earthquakes, as we do here, demonstrates the robustness of the system to false alarms. Analyzing near field records is a troublesome issue for EEW; since a complete theoretical framework is yet to be developed^57^, other approaches such as resolving line sources^58^ or extrapolating peak ground motions away from the earthquake source^59^ may need to be adapted to DAS data in order to address this gap.

The derivation of the presented physics-based magnitude estimation approach did not require any earthquake observations, a significant advantage since the scarcity of DAS earthquake observations hinders the derivation of empirical methods. Because no earthquakes were required, the approach is geographically independent and readily applicable in any tectonic setting using both offshore and onland fibers and different DAS interrogator units, as demonstrated here using earthquakes from Greece, France and Chile. Earthquake observations are only required to map well-coupled fiber segments, although this objective may also be achieved using ambient noise^16^. Using segments that are not well-coupled may lead to either magnitude under-estimation, if strain amplitudes are weak, or over-estimation, if a segment is suspended and experiences strong vibrations due to cable-waves^60^. The approach allows for continuous update of magnitude and ground motion predictions, key for analyzing large earthquakes with long durations. In addition, using a holistic magnitude estimation and ground motion prediction that are derived from the same earthquake model reduces the impact of stress drop related magnitude biases on ground motion predictions and enhances the overall robustness of the system.

The computational costs of the presented approaches are low. While DAS acquisitions typically provide very large data volumes, for EEW, data can be largely down-sampled in both time and space, limiting both the volume of data and processing times. For instance, to obtain timely and robust magnitude estimates, it is sufficient to use preselected well-coupled fiber portions at spacings of several kilometers. For the purpose of this study, we analyzed 180 s long recordings of preselected DAS fiber segments (33 channels), down-sampled to ~ 20 Hz, in ~ 136 s using a Python code on an Intel Core i7 laptop with 32 GB RAM using a single thread. These computation times indicate that the method is valid for real-time. For future implementation as part of an operational EEW system, several aspects of the code can be optimized and run in parallel. For example, slant-stack computations, which are the most time-consuming (~ 4 s per channel), can be parallelized, in addition to computations for different fiber segments. The latter can also increase the number of fiber segments used for magnitude estimation.

For few earthquakes, strain amplitudes exhibited a small degree of saturation. Nevertheless, magnitude estimations still allow for reliable ground motion predictions (Fig. 4). This phenomenon is insufficiently reported and investigated in existing literature and needs to be quantified and addressed as it may affect the ability to analyze higher strain amplitudes and provide reliable warnings for larger earthquakes. DAS saturation needs to be studied along with DAS manufacturers to devise preprocessing and postprocessing methods in order to fully demonstrate the viability of DAS for EEW.

The framework presented in this study demonstrates the great potential of using DAS for EEW. The approaches presented here allow for easy, robust, and fast implementation of EEW using both offshore and onland optical fibers in any tectonic setting. Specifically, using existing ocean-bottom optical fibers, which are almost ubiquitous along subduction zones worldwide, provide a cheap and readily available EEW solution, especially for exposed developing countries, that will significantly enhance earthquake hazard mitigation capabilities.

## Methods

### The relation between earthquake source parameters and ground motions

For large earthquakes , i.e., when high-frequency attenuation is negligible, recorded in the far-field, ground displacements root-mean-squares (rms), *D*_*rms*_, and peak ground displacements (PGD) are mostly a function of the seismic moment, *M*_0_: ${D}_{rms}\propto PGD\propto {M}_{0}^{5/6}\Delta {\tau }^{1/6}$ while ground velocities rms, *V*_*rms*_, and PGV, and accelerations rms, *A*_*rms*_, and PGA, are also strongly influenced by the stress drop, Δτ: ${V}_{rms}\propto PGV\propto {M}_{0}^{1/2}\Delta {\tau }^{1/2}$ and ${A}_{rms}\propto PGA\propto {M}_{0}^{1/3}\Delta {\tau }^{2/3}$ ^24,40^. The proportionality between rms and peak ground motions stems from statistical theories^61^ and was observed by previous studies^40^. Note the different powers associated with *M*_0_ and Δτ. Thus, ground displacements serve as a better magnitude predictor compared to velocities or accelerations^32,35,40^.

### Real-time strain-rates to ground accelerations conversion

The slant-stack^62^ based strains to ground motions conversion scheme^37^ accounts for apparent phase velocity variations in both time and space. The conversion is applied for each DAS channel along the fiber using short, approximately linear, fiber segments. Here, this recently presented approach^37^ is modified and optimized for real-time performance. The semblance (coherency) as a function of apparent slowness *p*_*x*_ and time *t*, for a DAS channel located at *x*_0_ along the fiber, can be written as:

2 $$sem\left( {p_{x} , t} \right) = \frac{1}{L}\left\{ {\begin{array}{*{20}c} {\frac{{\left[ {\mathop \sum \nolimits_{j = - L}^{ - 1} g\left( {t + p_{x} \left( {x_{j} - x_{0} } \right)} \right)} \right]^{2} }}{{\mathop \sum \nolimits_{j = - L}^{ - 1} g\left( {t + p_{x} \left( {x_{j} - x_{0} } \right)} \right)^{2} }}} & {if\,\,p_{x} > 0} \\ {\frac{{\left[ {\mathop \sum \nolimits_{j = 1}^{L} g\left( {t + p_{x} \left( {x_{j} - x_{0} } \right)} \right)} \right]^{2} }}{{\mathop \sum \nolimits_{j = 1}^{L} g\left( {t + p_{x} \left( {x_{j} - x_{0} } \right)} \right)^{2} }}} & {if\,\,p_{x} < 0} \\ \end{array} } \right.,$$

where *L* is the number of DAS channels used for slowness estimation, *g*(*t*) is the DAS strain-rates time-series, and *x*_*j*_—*x*_0_ is the distance between station *j* and the reference channel (at *x*_0_). Equation (2) can be regarded as the causal slant-stack, where only data samples of *g*(*t*) that have already been recorded are considered.

The conversion procedure is performed as follows. For computational efficiency, recorded strain-rates are down-sampled to 20 Hz (or slightly higher, depending on the original signals’ sampling-rate). Data is lowpass filtered at 5 Hz using a 4-pole Butterworth filter to diminish high frequency instrumental noise. The applied down-sampling and filtering did not decrease the robustness of the conversion and subsequent magnitude estimation. The local slant-stack transform is applied using fiber segments of ~ 380 m length^37^, with channel spacings of ~ 20 m, skipping several channels for densely spaced measurements. The used fiber segments are long enough to resolve long seismic wavelengths with fast velocities of several km/s, and short enough so that seismic waves are coherent and fiber sections are approximately linear^37^. Semblance is calculated using 50 predefined slowness values, equally spaced between − 5 s/km and 5 s/km. At each *t*, the wavefield’s slowness is determined as the one with highest semblance. The produced slowness time-series is then smoothed by applying a causal moving-mean filter of 1 s to its absolute value. Strain-rates time-series are then divided by the slowness time-series to obtain ground accelerations, followed by an additional 5 Hz lowpass filter. Because we are eventually interested in the converted strain-rates’ rms, the slowness’ sign may be discarded (See “Magnitude estimation from bandlimited ground accelerations” in Methods).

### Magnitude estimation from bandlimited ground accelerations

We derive an expression for the rms of the ground accelerations using the commonly used Omega-squared source model^41^ describing the far-field body wave spectra (grey dashed curve in Supplementary Fig. 9). This derivation procedure follows that used by several previous studies^24,35,40,63–66^. The acceleration omega-squared model^41^ subject to high frequency attenuation^67^ (grey dotted curve in Supplementary Fig. 9) reads as:

3 $$\ddot{\Omega }\left( f \right) = \left( {2\pi f} \right)^{2} \frac{{\Omega_{0} }}{{1 + \left( {\frac{f}{{f_{0} }}} \right)^{2} }}e^{ - \pi \kappa f} ,$$

where *f*_0_ is the source corner frequency, Ω_0_ is the displacement low frequency spectral plateau, and *κ* is an attenuation parameter. Since strain-rates are lowpass filtered at 5 Hz, acceleration rms are calculated using Eq. (3) as ${A}_{rms}=\sqrt{\frac{2}{T}{\int }_{f=0}^{f=5}{\left|\ddot{\Omega }\left(f\right)\right|}^{2}df}$ (black dashed curve in Supplementary Fig. 9), where *T* is the data interval. The integral is solved and an analytic approximation is obtained (See Supplementary Note 1). The spectral parameters Ω_0_ and *f*_0_ are substituted with the seismic moment^23^ and stress drop^68^, respectively, via:

4a $$M_{0} = \Omega_{0} \frac{{4\pi \rho C^{3} R}}{{U_{\varphi \theta } F_{s} }},$$

4b $$\Delta \tau = \frac{7}{16}M_{0} \left( {\frac{{f_{0} }}{{kC_{S} }}} \right)^{3} ,$$

where *ρ* is the density at the source, *C* is the wave velocity at the source (*C*_*P*_ and *C*_*S*_ for P- and S-waves, respectively), *R* is the hypocentral distance, *U*_*φθ*_ is the average radiation pattern, *F*_*S*_ is the free-surface correction, and *k* is a phase-specific constant which also depends on the source model and rupture speed^42^. Equation (4b) is valid for a circular crack embedded in a homogeneous medium^68^. The resulting expression is:

5 $${A}_{rms}^{approx}={A}_{1}{M}_{0}^\frac{1}{3}{\Delta \tau }^\frac{2}{3}\sqrt{1-{e}^{-2{\alpha }_{m}}}\frac{1}{R\sqrt{\kappa T}\left(1+\frac{{A}_{2}^{2}{\kappa }^{2}{\left(\frac{\Delta \tau }{{M}_{0}}\right)}^\frac{2}{3}\sqrt{1-{e}^{-2{\alpha }_{m}}}}{h\left({\alpha }_{m}\right)}\right)},$$

where the superscript *approx* signifies approximate rms, ${A}_{1}=\frac{{U}_{\varphi \theta }{F}_{s}\sqrt{\pi }}{\rho {C}^{3}}{\left(\frac{16}{7}\right)}^\frac{2}{3}{\left(k{C}_{S}\right)}^{2}$, ${A}_{2}=\pi {\left(\frac{16}{7}\right)}^{1/3}k{C}_{S}$, $h\left({\alpha }_{m}\right)={e}^{-{\alpha }_{m}}\sqrt{\frac{1}{2}\left[-3-6{\alpha }_{m}-6{\alpha }_{m}^{2}-4{\alpha }_{m}^{3}-2{\alpha }_{m}^{4}+3{e}^{2{\alpha }_{m}}\right]}$ and ${\alpha }_{m}=5\pi \kappa$.

Equation (5) can be analytically solved for the seismic moment:

6 $${M}_{0}=\frac{1}{27{a}_{1}}{\left(\frac{{a}_{4}}{{2}^\frac{1}{3}}+\frac{{2}^\frac{1}{3}{a}_{2}^{2}}{{a}_{4}}+{a}_{2}\right)}^{3},$$

where ${a}_{1}={A}_{1}{\Delta \tau }^\frac{2}{3}\sqrt{1-{e}^{-2{\alpha }_{m}}}\frac{1}{R\sqrt{\kappa T}}$, ${a}_{2}={A}_{rms}$, ${a}_{3}=\frac{{A}_{rms}{A}_{2}^{2}\Delta {\tau }^\frac{2}{3}{\kappa }^{2}\sqrt{1-{e}^{-2{\alpha }_{m}}}}{h\left({\alpha }_{m}\right)}$ and ${a}_{4}={\left(3\sqrt{3\left(27{a}_{1}^{4}{a}_{3}^{2}+4{a}_{1}^{2}{a}_{2}^{3}{a}_{3}\right)}+27{a}_{1}^{2}{a}_{3}+2{a}_{2}^{3}\right)}^\frac{1}{3}$. The moment magnitude can then be written as:

7 $${M}_{W}=2\left(\frac{{a}_{4}}{{2}^\frac{1}{3}}+\frac{{2}^\frac{1}{3}{a}_{2}^{2}}{{a}_{4}}+{a}_{2}\right) -\frac{2}{3}\left({a}_{1}\right) -7.05,$$

where *M*_0_ is expressed in Nm.

While the coefficients ${a}_{1}$, ${a}_{2}$, ${a}_{3}$ and ${a}_{4}$ contain many parameters, only few are updated in real-time: *A*_*rms*_ is continuously updated as new data is recorded, the available data interval *T* begins at the P-wave arrival and increases with time, and *R* is updated as earthquake location improves. The parameters used are^24^: *F*_*S*_ = 2, *ρ* = 2600 kg/m^3^, *C*_*S*_ = 3.2 km/s, *C*_*P*_ = 5.3 km/s, *κ* = 0.025 s, *U*_*φθ*_ equals 0.52 and 0.63 for P- and S-waves, respectively^23^, and *k* equals 0.32 and 0.21 for P-and S-waves, respectively^42^. For data intervals that contain both P- and S-waves, the phase specific constants need to be averaged based on the relative intervals of each phase^24^:

8 $$const=\frac{{T}_{S-P}}{T}cons{t}_{P}+\frac{T-{T}_{S-P}}{T}cons{t}_{S},$$

where *const* stands for *U*_*φθ*_, *C* or *k* for P- or S-waves, and *T*_*S-P*_ is the S-P data interval. Using these parameters, ${a}_{1}$ and ${a}_{3}$ may be written as:

9a $${a}_{1}=113014\left(\frac{{k}^{2}{U}_{\varphi \theta }}{{C}^{3}}\right){\Delta \tau }^\frac{2}{3}\frac{1}{R\sqrt{T}},$$

9b $${a}_{3}=1828968\left({k}^{2}\right)\Delta {\tau }^\frac{2}{3}{A}_{rms},$$

where phase-specific terms are written in parentheses.

In this application, the magnitude is estimated using several manually identified well-coupled fiber segments of ~ 600 m as follows. Strain-rates within each fiber segment are converted to ground accelerations (See “Real-time strain-rates to ground accelerations conversion” in Methods). *A*_*rms*_ is calculated per DAS channel starting at the P-wave arrival, and is then logarithmically averaged per fiber segment at every time-step to minimize the impact of outliers. Since DAS can only measure the wavefield in-line with the fiber, *A*_*rms*_ is multiplied by $\sqrt{2}$ to compensate for the missing orthogonal component. The averaged *A*_*rms*_ at time *T* is then input to Eq. (7) along with Δ*τ* and *R* to estimate the magnitude. Magnitude estimates are continuously updated until either averaged *A*_*rms*_ reaches its maximum value, or *T* = 60 seconds^24^. Magnitude estimates from different fiber segments are weight-averaged by the available data interval to obtain an event specific estimate.

### Ground motion prediction

For PGV and PGA prediction, we use a set of physics-based GMPEs^24,40^, derived using the same source model^41^ (Eq. 3) used to obtain the real-time magnitude expression (Eq. 7) (See “Magnitude estimation from bandlimited ground accelerations” in Methods). The GMPEs for PGV and PGA are:

10a $$PGV=2.9\sqrt{{M}_{0}\Delta \tau }\frac{{\beta }_{V}}{R\sqrt{\frac{1}{k{C}_{S}}{\left(\frac{7}{16}\frac{{M}_{0}}{\Delta \tau }\right)}^{1/3}+R/{C}_{S}}{\left[1+{\pi }^\frac{4}{3}{\kappa }_{0}k{C}_{S}{\left(\frac{16}{7}\frac{\Delta \tau }{{M}_{0}}\right)}^\frac{1}{3}\right]}^\frac{3}{2}},$$

10b $$PGA=3.3{M}_{0}^{1/3}{\Delta \tau }^{2/3}\frac{{\beta }_{A}}{R\sqrt{{\kappa }_{0}\left[\frac{1}{k{C}_{S}}{\left(\frac{7}{16}\frac{{M}_{0}}{\Delta \tau }\right)}^\frac{1}{3}+\frac{R}{{C}_{S}}\right]}{\left[1+{1.5}^{-\frac{1}{4}}\pi {\kappa }_{0}k{C}_{S}{\left(\frac{16}{7}\frac{\Delta \tau }{{M}_{0}}\right)}^\frac{1}{3}\right]}^{2}},$$

where ${\beta }_{V}=\frac{2\pi {U}_{\phi \theta }Fs\sqrt{\frac{16}{7}} {\left(k{C}_{S}\right)}^\frac{3}{2}}{(\sqrt{2\pi }4\rho {C}_{S}^{3})}$ and ${\beta }_{A}=\frac{4\pi {U}_{\phi \theta }Fs{\left(\frac{16}{7}\right)}^{2/3}{\left(k{C}_{S}\right)}^{2}}{\left(\sqrt{\pi }4\rho {C}_{S}^{3}\right)}$. These theoretical GMPEs are readily applicable in any seismic region. Using the parameter tuning for S-waves (See “Magnitude estimation from bandlimited ground accelerations” in Methods), *β*_*V*_ = 2.44 × 10^–10^ m^1.5^s^1.5^/kg and *β*_*A*_ = 2.05 × 10^–8^ m^2^s/kg.

### Synthetic ground motions

The GMPEs in Eq. (10) are used to generate synthetic PGV and PGA for different seismic moments, stress drops and hypocentral distances. Synthetic *A*_*rms*_ are generated by calculating the rms of the acceleration spectra (Supplementary Fig. 9). These spectra are produced for a specific seismic moment, stress drop and hypocentral distance using Eq. (3) and (4), subject to a lowpass filter. The filter is modeled in two manners: as a clean cutoff (dashed black curve in Supplementary Fig. 9) as used for the model derivation (See “Magnitude estimation from bandlimited ground accelerations” in Methods), or as an ideal 4-pole Butterworth filter (solid black curve in Supplementary Fig. 9), similar to that used for DAS signal processing.

### Earthquake dataset

DAS measurements in Greece were conducted using a Febus A1 DAS interrogator between 18–19 and 19–25 April 2019 on 13.2 km and 26.2 km long fibers, sampled at 6 ms and 5 ms, respectively. Gauge length and spatial sampling were both set to 19.2 m for the two fibers. DAS measurements in France were conducted using an Aragon Photonics hDAS interrogator between 11–31 July 2019 on a 44.8 km long fiber, sampled at 10 ms and 2 ms for the first and last 10 days, respectively. Gauge length and spatial sampling were both set to 10 m. DAS measurements in Chile were conducted using an ASN OptoDAS interrogator between 27 October and 3 December 2021 on a 204 km long fiber, sampled at 8 ms. Gauge length and spatial sampling were both set to 4.085 m. The Febus and OptoDAS interrogators record strain-rates while the Aragon instrument records stains; the latter were differentiated to strain-rates before the conversion to ground accelerations.

Seismometer and accelerometer recordings were used to calculate PGV and PGA for the different earthquakes as follows. Data for Greece, France and Chile were obtained from the National Observatory of Athens, the RESIF repository and IRIS, respectively. The two horizontal components of point-sensors were demeaned and highpass filtered at 1 Hz using a 4-pole Butterworth filter, followed by a simple gain correction. Velocity-meter signals were differentiated to obtain ground accelerations and accelerometer records were integrated to obtain ground velocities. An additional highpass filter was applied after differentiations and integrations. PGV (PGA) were then calculated as the geometric mean of the maximum of the absolute value of the two velocity (acceleration) components. PGV and PGA that are smaller than 5 times the standard deviation of the associated time-series are discarded as they may be biased by noise.

## Supplementary Information

Supplementary Information.

## Data Availability

Samples of DAS earthquakes are available on https://osf.io/4bjph/.
