# Supplementary material for: Magnitude estimation and ground motion prediction to harness fiber optic distributed acoustic sensing for earthquake early warning
Source: Sci Rep. 2023 Jan 9;13:424. doi: 10.1038/s41598-023-27444-3 (PMC9829724; doi:10.1038/s41598-023-27444-3)
Supplement: Supplementary file 1 — Supplementary Information. [file 41598_2023_27444_MOESM1_ESM.pdf]

Supplementary Information for

Magnitude Estimation and Ground Motion Prediction to Harness Fiber Optic Distributed Acoustic Sensing for Earthquake Early Warning

Itzhak Lior[1], Diane Rivet[2], Jean-Paul Ampuero[2], Anthony Sladen[2], Sergio Barrientos[3], Rodrigo Sánchez-Olavarría[3], German Alberto Villarroel Opazo[4], Jose Antonio Bustamante Prado[4]

[1]Institute of Earth Sciences, The Hebrew University, Jerusalem, Israel

[2]Université Côte d'Azur, Observatoire de la Côte d'Azur, CNRS, IRD, Géoazur, France

[3]Centro Sismológico Nacional, Universidad de Chile, Santiago, Chile

[4]Gtd Grupo S.A.,Santiago, Chile

Contents of this file

Supplementary Note 1: Bandlimited ground accelerations rms derivation

Supplementary Note 2: The effect of stress drop variability on magnitude, PGV and PGA predictions

Supplementary Figure 1: Example of an earthquake recorded along 150km of fiber

Supplementary Figure 2: Maps

Supplementary Figure 3: Strain-rates to ground accelerations conversion and magnitude estimation for a magnitude 5.7 earthquake

Supplementary Figure 4: Comparison between strain-rates to ground accelerations conversions

Supplementary Figure 5: Magnitude and distance distribution

Supplementary Figure 6: Real-time magnitude estimation and ground motion prediction using 1 MPa

Supplementary Figure 7: Real-time magnitude estimation and ground motion prediction using 10 MPa

Supplementary Figure 8: Average magnitude, and PGV and PGA residuals for a magnitude 5.7 recorded offshore Chile

Supplementary Figure 9: Earthquake source model

Supplementary Table 1: Earthquake catalog

Supplementary Note 1: Bandlimited ground accelerations rms derivation

we first perform a change of variables by defining $\alpha = \pi\kappa f$, $\alpha_0 = \pi\kappa f_0$ and $\alpha_m = \pi\kappa f_{max}$. Eq. (3) in the main text may be written as:

$$\ddot{\Omega}(f) = (2\alpha)^2 \frac{1}{\kappa^2} \frac{\Omega_0}{1+\left(\frac{\alpha}{\alpha_0}\right)^2} e^{-\alpha}. \tag{S1}$$

calculating the root-mean-squares (rms):

$$A_{rms} = \sqrt{\frac{2}{T}\int_{f=0}^{f=5} \left|\ddot{\Omega}(f)\right|^2 df}. \tag{S2}$$

Inserting Eq. (S1) into (S2) we obtain:

$$A_{rms} = \sqrt{\frac{2}{\pi\kappa T}}\,\Omega_0 \frac{4}{\kappa^2} \sqrt{\frac{\alpha_0^4}{4}\left[2 - \frac{2(\alpha_m^2+\alpha_0^2-\alpha_m\alpha_0^2)e^{-2\alpha_m}}{\alpha_f^2+\alpha_0^2} + \alpha_0(-3i+2\alpha_0)e^{2i\alpha_0}Ei(-2 \right.} \tag{S3}$$

,

where Ei is the exponent integral function. Since α_0 is proportional to f_0, a measure of the earthquake's duration and size[47,54], α_0 is used to find the asymptotic limits of Equation (S3) for large earthquakes:

$$A_{rms} = \Omega_0\alpha_0^2 \frac{4}{\kappa^2\sqrt{\pi\kappa T}}\sqrt{1-e^{-2\alpha_m}}, \tag{S4a}$$

and small earthquakes:

$$A_{rms} = \Omega_0 \frac{4}{\kappa^2\sqrt{\pi\kappa T}} h(\alpha_m), \tag{S4b}$$

where $h(\alpha_m) = e^{-\alpha_m}\sqrt{\frac{1}{2}(-3-6\alpha_m-6\alpha_m^2-4\alpha_m^3-2\alpha_m^4+3e^{2\alpha_m})}$.

An analytic approximation that satisfies both asymptotic limits is:

$$A_{rms}^{approx} = \frac{4}{\kappa^2\sqrt{\pi\kappa T}}\Omega_0\sqrt{1-e^{-2\alpha_m}} \frac{\alpha_0^2}{1+\frac{\alpha_0^2\sqrt{1-e^{-2\alpha_m}}}{h(\alpha_m)}}. \tag{S5}$$

Eq. (S5) is then expressed in terms of seismic moment, M_0, and stress drop, $\Delta\tau$, in Eq (5) in the main text.

Supplementary Note 2: The effect of stress drop variability on magnitude, PGV and PGA predictions

Magnitude, PGV and PGA residuals exhibit complex non-intuitive patterns in Fig. (3). We summarize here their characteristics and origins.

- Panel a: Small earthquakes' ground motions, and magnitude estimates, are insensitive to stress drop[36] (See "Ground motion prediction" in Methods), resulting in identical estimates for different values. For large earthquakes, $A_{rms} \propto M_0^{1/3} \Delta\tau^{2/3}$ (See "The relation between earthquake source parameters and ground motions" in Methods). Because magnitude estimates are based on A_{rms} observations, for a given A_{rms}, changing the stress drop by an order of magnitude will change M_0 estimates by 2 orders of magnitude, and magnitude estimates by 4/3 magnitude units (note that $M_W \propto \frac{2}{3} log\, log\, (M_0)$.
- Panels c, e: Because the ground motions of small magnitude earthquakes are invariant to stress drop, all curves are near 0. For large earthquakes $PGV \propto M_0^{1/2} \Delta\tau^{1/2}$ and $PGA \propto M_0^{1/3} \Delta\tau^{2/3}$ (See "The relation between earthquake source parameters and ground motions" in Methods), where rms measures are proportional to their peak values[36]. When predicting PGV and PGA using the synthetic magnitude and a stress drop that is biased by an order of magnitude, PGV and PGA are biased by up to half an order magnitude and 2/3 order of magnitude respectively (dashed curves in panels c, e). However, when the predicted magnitude is used along with an over-estimated (under-estimated) stress drop, PGV should have been under-estimated (over-estimated) by up to half an order of magnitude and PGA should have been 0. The shapes of the curves in panels (c, e) differ from the described behavior because stress drop has additional second order contributions to PGV and PGA predictions (See "Ground motion prediction" in Methods), i.e., in the denominators of Eq. (10), and because of the approximations made in the derivation of the magnitude estimation expression (See Supplementary Note 1).

- Panels b, d, f: When stress drop is known, residuals are small and non-zero around magnitude 4, a result of the approximations made in the derivation of the magnitude estimation expression (See Supplementary Note 1).
- All panels: At small magnitudes, discrepancies are non-zero for all possible stress drops, a result of the difference between the way A_{rms} is calculated (See "Synthetic ground motions" in Methods): Using a clean cut-off lowpass filter for model derivation (dashed black curve in Supplementary Fig. 8) and using a 4-pole Butterworth lowpass filter when analyzing observations (solid black curve in Supplementary Fig. 8). This discrepancy is most prominent for small earthquakes

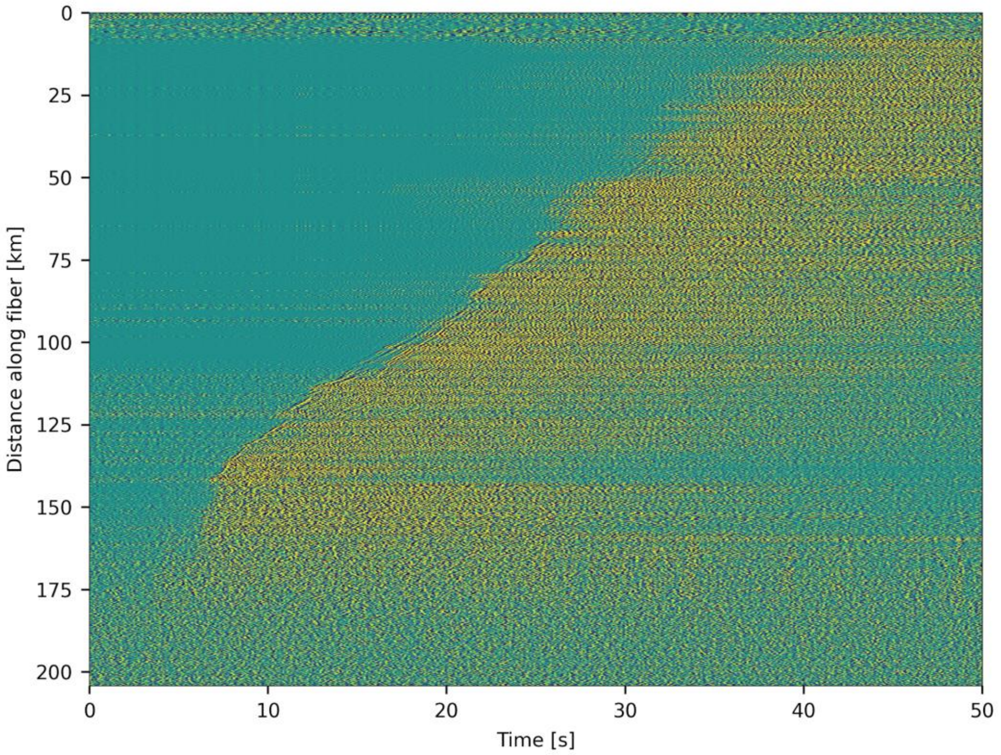

Supplementary Figure 1: Example data recording of an Earthquake in Chile. A magnitude 3.8 earthquake recorded at hypocentral distances between 35 km (at 150 km along the fiber) and 150 km (at the interrogator) by an Alcatel OptoDAS interrogator unit. Data are bandpass filtered between 1 and 5 Hz and each channel along the fiber is normalized by its standard deviation. The earthquake signal is observed with high fidelity up to at least 150 km along the fiber.

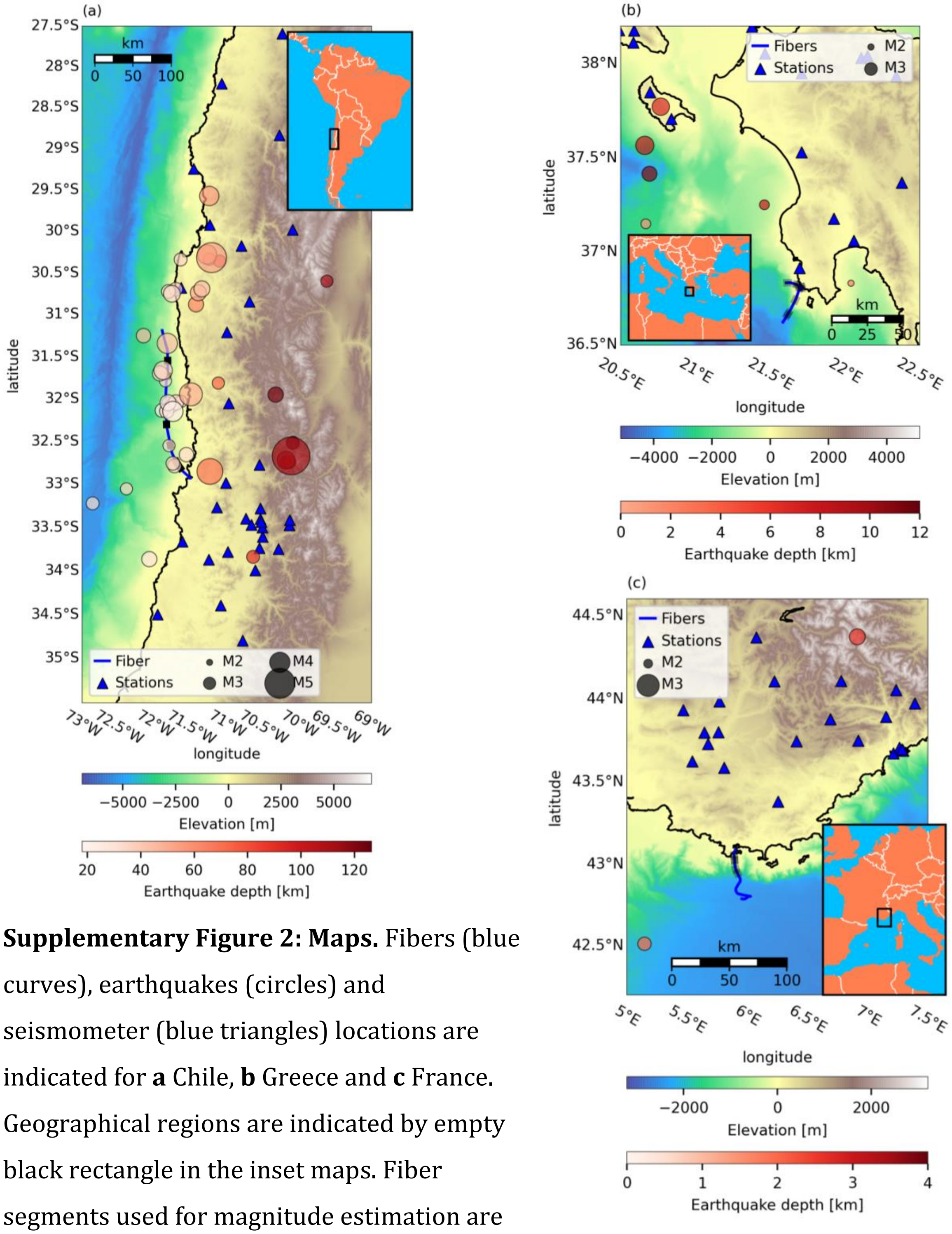

Supplementary Figure 2: Maps. Fibers (blue curves), earthquakes (circles) and seismometer (blue triangles) locations are indicated for **a** Chile, **b** Greece and **c** France. Geographical regions are indicated by empty black rectangle in the inset maps. Fiber segments used for magnitude estimation are indicated by solid black rectangles. Color code corresponds to the topography and circle color code corresponds to earthquake depth. Maps were generated using Python's Basemap package and bathymetric data downloaded from ncei.noaa.gov/maps/bathymetry/.

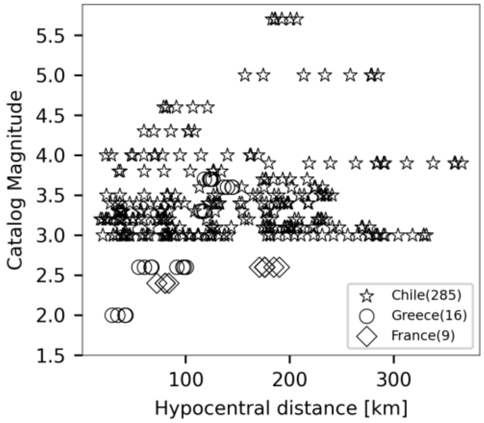

Supplementary Figure 3: Magnitude and distance distribution. Catalog magnitudes as a function of hypocentral distances for DAS recorded earthquakes. Each data point corresponds to an earthquake recorded by one (of several) fiber segments. Earthquakes from Chile, Greece and France are indicated by stars, circles and triangles, respectively, and the number of points is indicated in the legend.

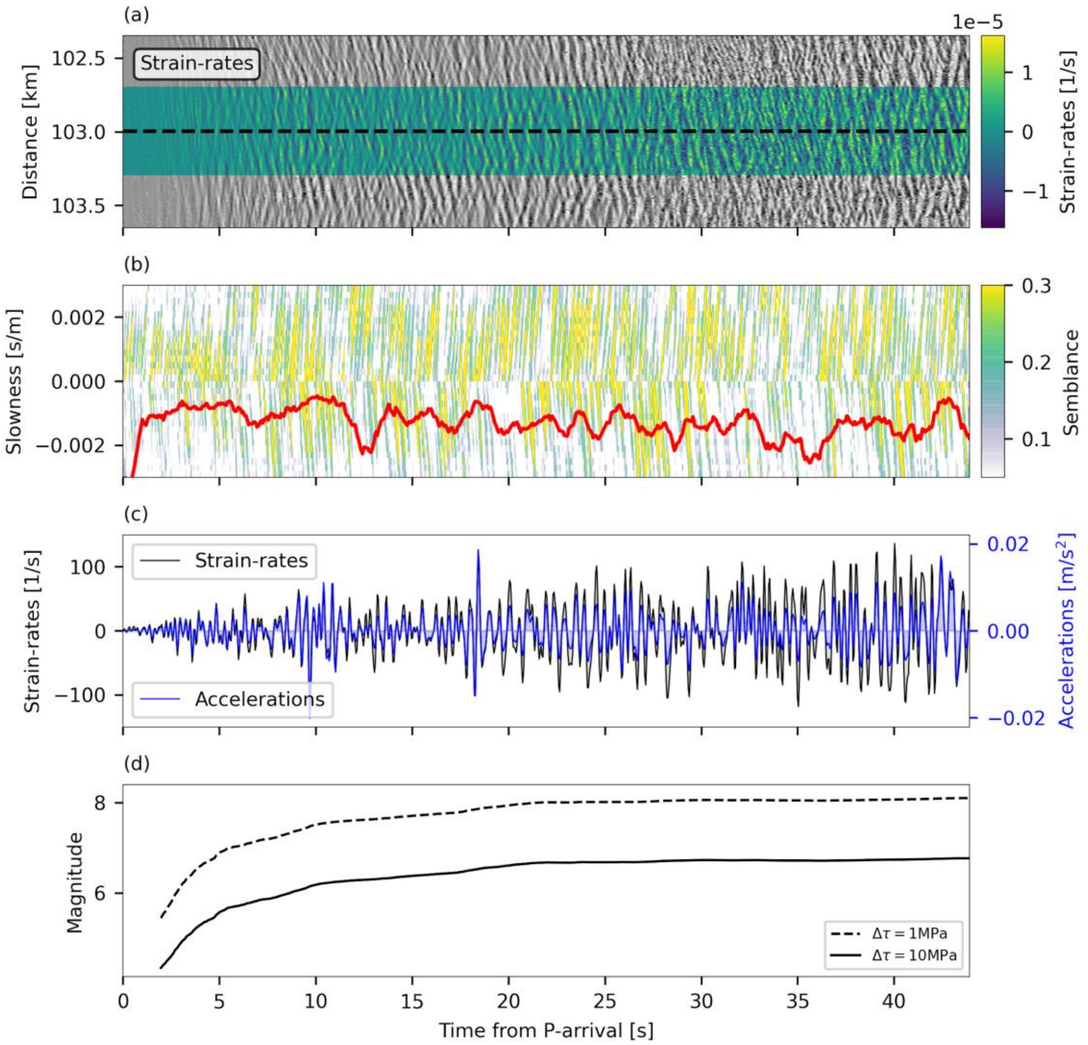

Supplementary Figure 4: Strain-rates to ground accelerations conversion and magnitude estimation for a magnitude 5.7 earthquake. a Strain-rates recorded at a hypocentral distance of 207 km between 102.3 km and 103.6 km along a fiber offshore Chile. The fiber segment used for magnitude estimation is color-coded (102.7 km to 103.3 km). **b** Semblance as functions of apparent slowness and time from P-wave arrival for a reference DAS channel at 103 km from the interrogator (black dashed line in **a**). Smoothed slowness (See Methods) is indicated by a red curve. **c** Strain-rates (black) and converted ground accelerations (blue) for the reference DAS channel. **d** Real-time magnitude evolution using stress drops of 1 MPa (dashed curve) and 10 MPa (solid curve).

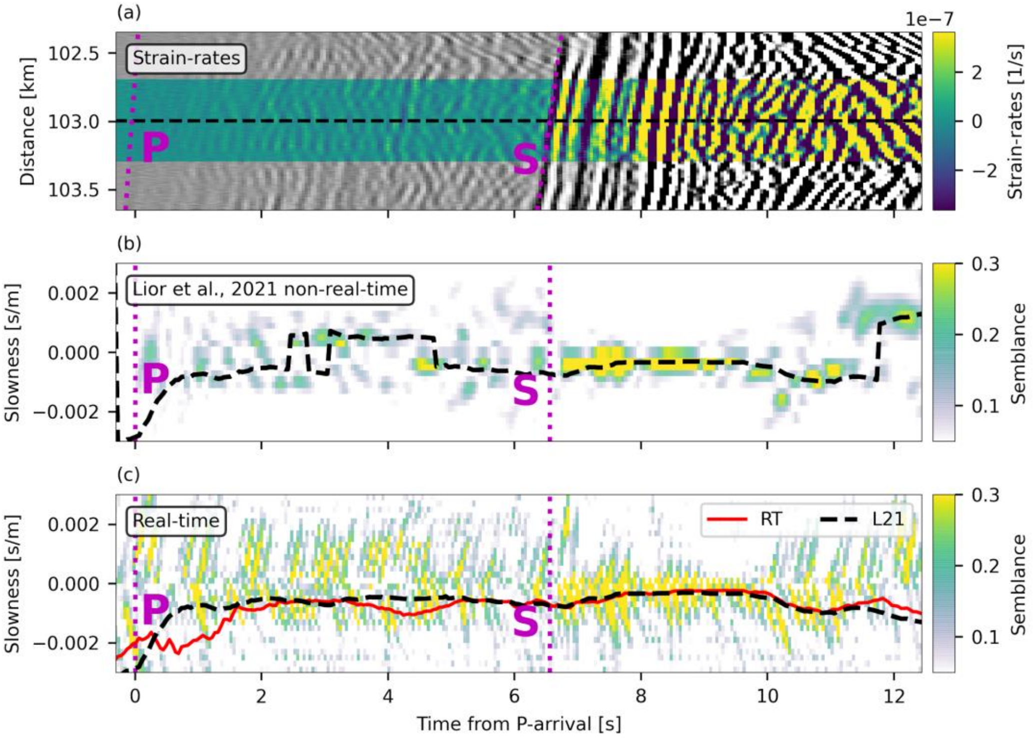

Supplementary Figure 5: Comparison between strain-rates to ground accelerations conversions. **a** Strain-rates of a magnitude 3.8 earthquake recorded at a hypocentral distance of 60 km between 102.3 km and 103.6 km along a fiber offshore Chile. Semblance as functions of apparent slowness and time from P-wave arrival for a reference DAS channel at 103 km from the interrogator (black dashed line in **a**) for **b** the recently proposed non-real-time conversion[34] and **c** the real-time conversion. Smoothed slowness (See Methods) is indicated by a black dashed curve **b** and red curve **c**. The absolute value of the non-real-time slowness (black dashed curve) is compared with the real-time slowness **c**. Manually picked P- and S-wave arrival times are indicated by magenta lines.

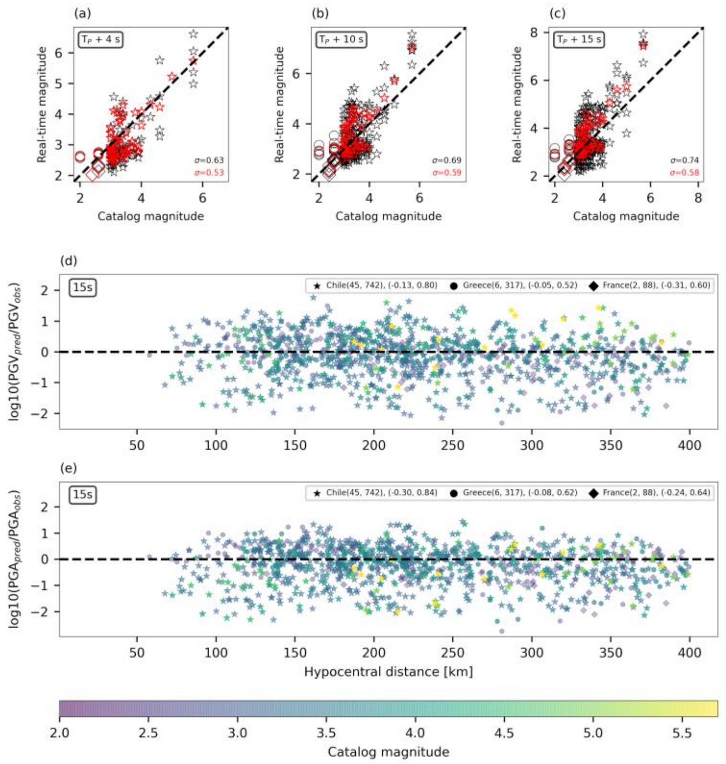

Supplementary Figure 6: Real-time magnitude estimation and ground motion prediction using 1 MPa. Real-time magnitude as a function of catalog magnitude at **a** 4, **b** 10 and **c** 15 seconds from the P-wave arrival at the first fiber segment using 1 MPa (Eq. 7 and 10). Fiber-segment-specific estimates and event averages are indicated by black and red symbols, respectively. The dashed black line is a 1:1 line and the standard deviations of the magnitude residuals are indicated in the bottom right corners for segment specific (black) and event averaged (red) estimates. Discrepancies between the logarithms of predicted and observed peak ground motions are plotted for **d** PGV and **e** PGA as functions of hypocentral distance. Color-code corresponds to catalog magnitudes. Earthquakes from Chile, Greece and France are indicated by stars, circles and triangles, respectively. Panel legends indicate the following: cable name (number of earthquakes, number of PGV and PGA observations), (average residuals, standard deviation to the residuals). Average within event variabilities, i.e., the optimal standard deviation to the residuals, for PGV are 0.68, 0.5 and 0.52 for Chile, Greece and France, respectively, and for PGA are 0.71, 0.61 and 0.59 for Chile, Greece and France, respectively.

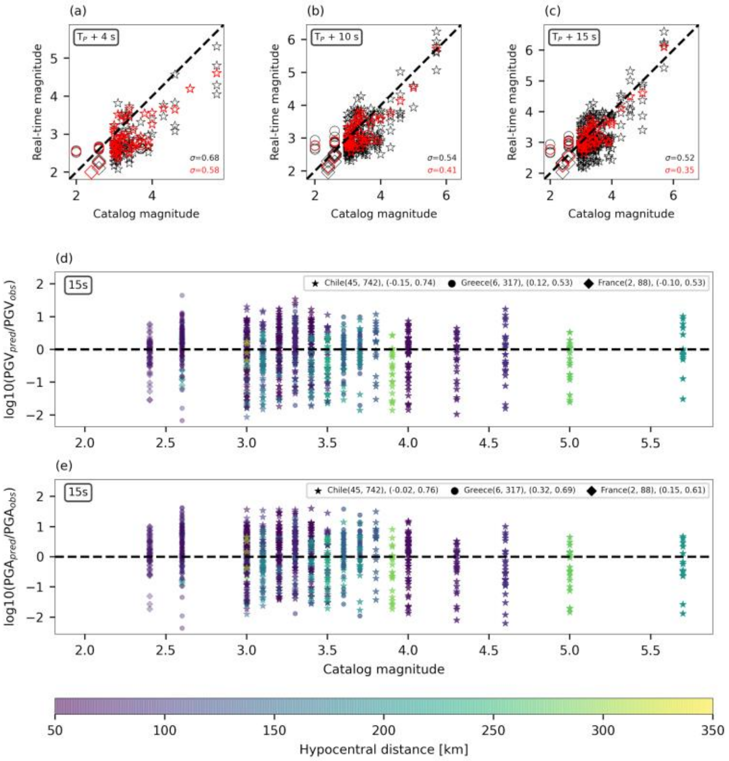

Supplementary Figure 7: Real-time magnitude estimation and ground motion prediction using 10 MPa. Real-time magnitude as a function of catalog magnitude at **a** 4, **b** 10 and **c** 15 seconds from the P-wave arrival at the first fiber segment. Fiber-segment-specific estimates and event averages are indicated by black and red symbols, respectively. The dashed black line is a 1:1 line and the standard deviations of the magnitude residuals are indicated in the bottom right corners for segment specific (black) and event averaged (red) estimates. Discrepancies between the logarithms of predicted and observed peak ground motions are plotted for **d** PGV and **e** PGA as functions of catalog magnitude. Color-code corresponds to hypocentral distances. Earthquakes from Chile, Greece and France are indicated by stars, circles and triangles, respectively. Panel legends indicate the following: cable name (number of earthquakes, number of PGV and PGA observations), (average residuals, standard deviation to the residuals). Average within event variabilities, i.e., the optimal standard deviation to the residuals, for PGV are 0.68, 0.5 and 0.52 for Chile, Greece and France, respectively, and for PGA are 0.71, 0.61 and 0.59 for Chile, Greece and France, respectively.

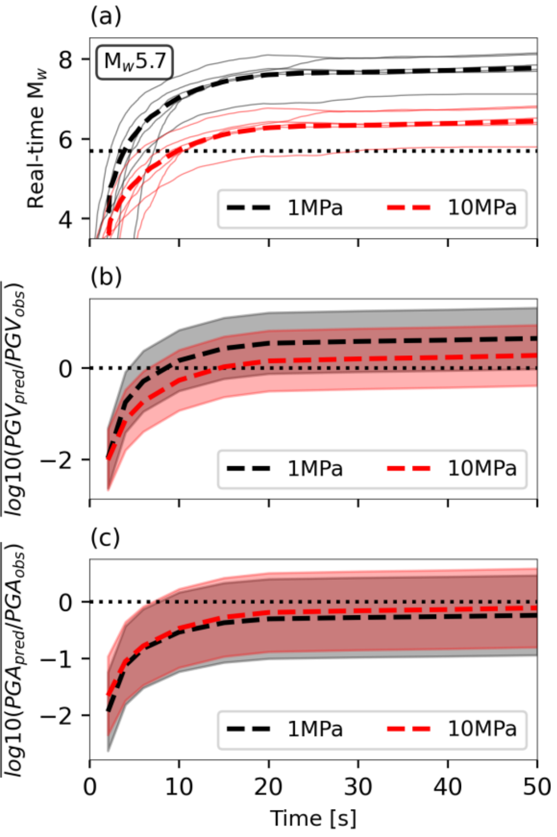

Supplementary Figure 8: Average magnitude, and PGV and PGA residuals for a magnitude 5.7 recorded offshore Chile. a Fiber-segment-specific (thin curves) and event averaged (thick dashed curves) magnitude using 1 MPa (black) and 10 MPa (red). Catalog magnitude is indicated by a dotted line. The average discrepancies between the logarithms of predicted and observed peak ground motions are plotted for **b** PGV and **c** PGA as functions of time from the P-wave arrival at the first fiber segment. Average residuals are plotted in black (1 MPa) and red (10 MPa) dashed curves and the standard deviations are indicated by semi-transparent regions of the same color. Zero residuals are indicated by dotted lines.

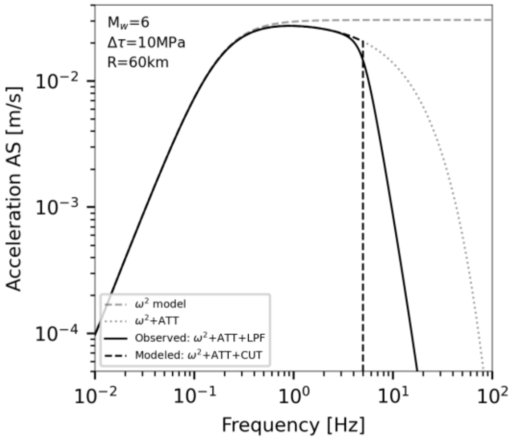

Supplementary Figure 9: Earthquake source model. The omega-squared model plotted for S-waves of a magnitude 6 earthquake with a 10 MPa stress drop at a hypocentral distance of 60 km, is indicated by a grey dashed curve. The model subject to high-frequency attenuation (ω^2+ATT) is indicated by a grey dotted curve. The model subject to high-frequency attenuation and a lowpass filter is indicated by a solid black curve using an ideal 4-pole Butterworth filter (ω^2+ATT+LPF), and a dashed black curve using a clean cutoff (ω^2+ATT+CUT).

Supplementary table 1: Earthquakes used in this study

Magnitude	Latitude	Longitude	Depth [km]	UTC time	Region
2 Ml	36.8335	22.0382	2	21/04/2019 22:11	Greece
2.4 Ml	42.516	5.143	2	21/07/2019 23:01	France
2.6 Ml	37.2528	21.4593	9	23/04/2019 19:25	Greece
2.6 Ml	37.1523	20.6662	1	19/04/2019 03:30	Greece
2.6 Ml	44.374	6.913	2.6	19/07/2019 21:16	France
3.0 Ml	-33.043	-72.393	35.1	20/11/2021 18:20	Chile
3.0 Ml	-31.779	-71.852	31.3	15/11/2021 14:17	Chile
3.0 Ml	-30.353	-71.102	86.1	14/11/2021 23:28	Chile
3.0 Ml	-32.046	-71.783	20	13/11/2021 02:05	Chile
3.0 Ml	-31.686	-71.901	38.9	10/11/2021 22:06	Chile
3.0 Ml	-32.021	-71.638	23	08/11/2021 12:36	Chile
3.0 Ml	-32.538	-71.803	31.8	06/11/2021 13:00	Chile
3.0 Ml	-30.329	-71.244	52.3	04/11/2021 18:26	Chile
3.0 Ml	-32.731	-70.187	114.2	04/11/2021 09:22	Chile
3.0 Ml	-30.592	-69.62	113.4	03/11/2021 02:48	Chile
3.0 Ml	-31.804	-71.119	72.4	30/10/2021 23:18	Chile
3.1 Ml	-33.833	-70.641	91.8	17/11/2021 13:21	Chile
3.1 Ml	-32.039	-71.814	37.8	04/11/2021 04:46	Chile
3.1 Ml	-30.334	-71.641	35.6	03/11/2021 07:52	Chile
3.1 Ml	-32.733	-70.14	102.4	03/11/2021 07:08	Chile
3.1 Ml	-33.213	-72.861	31.9	29/10/2021 10:59	Chile
3.1 Ml	-32.507	-70.091	126.4	29/10/2021 06:52	Chile
3.2 Ml	-32.126	-71.903	18.1	22/11/2021 10:28	Chile
3.2 Ml	-30.689	-71.708	35.5	19/11/2021 23:19	Chile
3.2 Ml	-32.066	-71.758	19.1	19/11/2021 00:49	Chile
3.2 Ml	-30.717	-71.811	32.9	18/11/2021 10:03	Chile
3.2 Ml	-32.746	-71.749	37.2	17/11/2021 07:11	Chile
3.2 Ml	-32.778	-71.726	23.3	15/11/2021 15:17	Chile
3.3 Ml	-32.135	-71.833	35.9	21/11/2021 22:57	Chile
3.3 Ml	-31.243	-72.155	45.1	15/11/2021 23:48	Chile
3.3 Ml	-32.646	-71.561	33.4	14/11/2021 23:13	Chile
3.3 Ml	37.4185	20.6897	11	22/04/2019 19:26	Greece
3.4 Ml	-31.94	-70.328	122.3	18/11/2021 06:49	Chile
3.4 Ml	-32.031	-71.704	34.7	13/11/2021 01:40	Chile
3.4 Ml	-33.856	-72.074	28.1	13/11/2021 00:24	Chile
3.4 Ml	-31.689	-71.928	27.2	10/11/2021 15:07	Chile
3.4 Ml	-30.872	-71.43	62.1	30/10/2021 01:49	Chile
3.5 Ml	-30.687	-71.347	43.5	24/11/2021 04:01	Chile
3.5 Ml	-30.732	-71.397	50.1	04/11/2021 12:40	Chile
3.5 Ml	-32.036	-71.816	24	03/11/2021 22:13	Chile

3.6 Ml	-30.744	-71.763	33.1	18/11/2021 09:24	Chile
3.6 Ml	37.7753	20.7658	7	23/04/2019 17:29	Greece
3.7 Ml	-32.71	-70.187	107.5	25/11/2021 03:18	Chile
3.7 Ml	37.57	20.66	8	18/04/2019 21:44	Greece
3.8 Ml	-31.655	-71.885	35.9	12/11/2021 20:35	Chile
3.9 Ml	-29.57	-71.246	55	18/11/2021 18:22	Chile
3.9 Ml	-30.271	-71.276	62.6	30/10/2021 15:01	Chile
4.0 Ml	-32.138	-71.749	22.3	16/11/2021 20:21	Chile
4.0 Ml	-31.334	-71.827	43.2	01/11/2021 13:43	Chile
4.3 Ml	-31.938	-71.502	48.7	24/11/2021 21:53	Chile
4.6 Mw	-32.839	-71.239	69	10/11/2021 06:51	Chile
5.0 Mw	-30.31	-71.216	52.2	04/11/2021 04:18	Chile
5.7 Mww	-32.661	-70.115	113	03/11/2021 03:17	Chile
